# Annual Research Review: Transdiagnostic neuroscience of child and adolescent mental disorders – differentiating decision making in attention‐deficit/hyperactivity disorder, conduct disorder, depression, and anxiety

**DOI:** 10.1111/jcpp.12496

**Published:** 2015-12-26

**Authors:** Edmund J. S. Sonuga‐Barke, Samuele Cortese, Graeme Fairchild, Argyris Stringaris

**Affiliations:** ^1^ Developmental Brain‐Behaviour LaboratoryAcademic Unit of PsychologyUniversity of SouthamptonSouthamptonUK; ^2^ Child Study Center at NYU Langone Medical CenterNew YorkNYUSA; ^3^ Institute of Psychiatry, Psychology and NeuroscienceKing's College LondonLondonUK

**Keywords:** Transdiagnostic, decision making, reinforcement learning, delayed reinforcement, executive functions, working memory, inhibition, prefrontal cortex, ventral striatum, amygdala: CD, attention‐deficit/hyperactivity disorder, anxiety, depression

## Abstract

**Background:**

Ineffective decision making is a major source of everyday functional impairment and reduced quality of life for young people with mental disorders. However, very little is known about what distinguishes decision making by individuals with different disorders or the neuropsychological processes or brain systems underlying these. This is the focus of the current review.

**Scope and methodology:**

We first propose a neuroeconomic model of the decision‐making process with separate stages for the prechoice evaluation of expected utility of future options; choice execution and postchoice management; the appraisal of outcome against expectation; and the updating of value estimates to guide future decisions. According to the proposed model, decision making is mediated by neuropsychological processes operating within three domains: (a) self‐referential processes involved in autobiographical reflection on past, and prospection about future, experiences; (b) executive functions, such as working memory, inhibition, and planning, that regulate the implementation of decisions; and (c) processes involved in value estimation and outcome appraisal and learning. These processes are underpinned by the interplay of multiple brain networks, especially medial and lateralized cortical components of the default mode network, dorsal corticostriatal circuits underpinning higher order cognitive and behavioral control, and ventral frontostriatal circuits, connecting to brain regions implicated in emotion processing, that control valuation and learning processes.

**Findings and conclusion:**

Based on clinical insights and considering each of the decision‐making stages in turn, we outline disorder‐specific hypotheses about impaired decision making in four childhood disorders: attention‐deficit/hyperactivity disorder (ADHD), conduct disorder (CD), depression, and anxiety. We hypothesize that decision making in ADHD is deficient (i.e. inefficient, insufficiently reflective, and inconsistent) and impulsive (biased toward immediate over delayed alternatives). In CD, it is reckless and insensitive to negative consequences. In depression, it is disengaged, perseverative, and pessimistic, while in anxiety, it is hesitant, risk‐averse, and self‐deprecating. A survey of current empirical indications related to these disorder‐specific hypotheses highlights the limited and fragmentary nature of the evidence base and illustrates the need for a major research initiative in decision making in childhood disorders. The final section highlights a number of important additional general themes that need to be considered in future research.

## Introduction

Success or failure in life is partly determined by the decisions one makes. In this review, we argue that ineffective decision making contributes to impaired functioning and reduced life satisfaction in children and adolescents with mental health conditions. These individuals' propensity to make poor decisions, while strikingly apparent to clinicians and other professionals, is underresearched. Little is known about the neuropsychological mechanisms that underpin decision‐making deficits in child and adolescent mental disorders, and crucially, there has been no systematic attempt to understand how these processes and mechanisms might differ *between* disorders.

In this review, we adopt a neuroeconomic perspective on decision making to address these issues. This approach provides an alternative framework to traditional psychiatric models (Hasler, 2012; Kishida, King‐Casas, & Montague, 2010) and potentially offers new insights into the ways in which complex behavioral processes are compromised in those with mental disorders. From our perspective, the decision‐making process can be broken down into a number of stages. For instance, whether a teenage patient has the motivation to attend her schoolmate's party depends on her evaluation of whether she will derive pleasure from doing so (‘evaluation of the subjective utility of a future event’, in neuroeconomic language). This is distinct from implementing the decision to act – how she will go about organizing herself and her environment so she is able to attend the party. These two processes are also distinct from her appraisal of the outcome – whether or not her experiences of the party were positive, neutral, or negative, and how that altered her views of herself and the value she attributes to such social encounters. The different stages of decision making will be associated with different expressions of psychopathology: a depressed teenager may find it hard to motivate herself to go to the party, whereas someone with ADHD may find it hard to generate and follow through a plan to get there. In contrast, a person with anxiety might attend the party but spend most of it scrutinizing their own actions and worrying about how they are perceived by others.

It is important to note that such a neuroeconomic approach to decision‐making rests on the core assumption that each action an individual performs is in some sense a choice – whether or not it is recognized as such by the individual (Sonuga‐Barke & Fairchild, 2012). In the party example, even an unmotivated and disengaged or an anxious‐avoidant response to the invitation to the party reflects a choice. Another assumption is that each individual, including those with mental disorders, sets out to maximize *subjective* value or utility through their actions – whether or not that goal is actually achieved. It is important to understand here that maximizing subjective utility does not necessarily imply maximizing the actual benefits available to a person. In fact, using the example above, a decision not to attend a party can be seen as rational from an anxious adolescent's perspective given the negative utility they attach to incurring social embarrassment, but when viewed more objectively, this decision may be damaging at a number of levels (i.e. reduced social interaction and ability to develop coping strategies and exacerbation of anxiety due to avoidance).

Leaving aside the issue of mental health‐related differences in economic goals, there are also barriers to effective decision making that are associated with impairments at different stages of the decision‐making process: Even if an individual has the same goals, they may differ from other individuals in their ability to make and carry through decisions to achieve those goals. In this sense, effective choice depends on the individual's ability to compare the subjective utility that may be derived in the future from the different choice options available (Oppenheimer & Kelso, 2015). These options may differ in terms of their valence (gains or losses), timing (immediate or delayed outcomes), and risk/probability (likely or unlikely). Furthermore, decision making is informed by both state‐ and trait‐like characteristics. Instances of state‐level differences are (a) *intrinsic* intraindividual variations in motivational states linked to physiological drives and energetic factors (e.g. hunger, thirst, need for sleep; de Ridder, Kroese, Adriaanse, and Evers 2014); and (b) extrinsic variations in elements such as the quality and availability of information about alternative actions and their consequences (Newell & Shanks, 2014) or external pressure (Byrne, Silasi‐Mansat, & Worthy, 2015; Stringaris, 2015). Returning to the example given above, fatigue would be an intrinsic state factor, while knowledge about who might be at the party would be an extrinsic one. At the trait level, individuals vary from one another in (a) their hierarchy of tastes and preferences with regard to different choice outcomes (a factor related to variations in subjective value assignment; Plassmann, O'Doherty, & Rangel, 2010); and (b) the efficiency with which they can process choice‐related information and implement their decisions.

In the current review, the complexity and the multifaceted nature of putative decision‐making deficits in mental disorders will be explored by contrasting aspects of the neuropsychology and pathophysiology of four mental disorders affecting children and adolescents: depression, anxiety, attention‐deficit/hyperactivity disorder (ADHD), and conduct disorder (CD). Such transdiagnostic comparisons are timely given the growing emphasis on identifying core dimensions of pathophysiological impairment that are relevant across different clinical presentations. This perspective, although not new, is currently being promoted by the National Institute for Mental Health (NIMH) through their Research Domain Criteria (RDoC) initiative (Insel et al., 2010). The aim of RDoC is to shift the focus of research, and eventually clinical practice, away from existing diagnostic categories, as recently updated in the DSM‐5 (American Psychiatric Association, 2013), toward ‘new ways of classifying psychopathology based on dimensions of observable behavior and neurobiological measures.’ The objective is to ‘define basic dimensions of functioning … cutting across disorders as traditionally defined’ (NIMH, http://www.nimh.nih.gov/research-priorities/rdoc/index.shtml; Cuthbert & Insel, 2013). The RDoC emphasis on transdiagnostic approaches represents a positive move to refocus a scientific field increasingly fragmented into diagnostic specialisms. However, there is considerable debate about the merits of this approach (Peterson, 2015). In light of this imperative, we are particularly interested to see whether potentially diverse patterns of decision‐making impairment across mental disorders implicate similar neuropsychological and neurobiological systems.

We chose to focus on ADHD, CD, anxiety, and depression because (a) each is relatively common in childhood and adolescence (Polanczyk, Salum, Sugaya, Caye, & Rohde, 2015); (b) they frequently co‐occur (Kessler et al., 2005); and (c) they encompass a broad range of psychopathological dimensions, both internalizing and externalizing; in this latter sense, they provide a strong test of the value of transdiagnostic approaches. It is also important to note that clinical observation and laboratory‐based experimental research combine to suggest that problems with decision making are present in each disorder. Clinically, core features of the four disorders implicate decision‐making impairments. Individuals with ADHD are often described as disinhibited and impulsive – choosing without sufficient reflection and favoring immediacy over delayed outcomes. CD is linked with risk‐taking and failure to learn from negative consequences. In contrast, anxious individuals tend to be oversensitive to risk of negative outcomes, while individuals with depression may be characterized as generally unmotivated and indecisive. It is important to note that despite the accepted complexity and heterogeneity of each of the disorders and the related existence of different types and subtypes within current diagnostic manuals, we feel that the purposes of our analysis are, at this stage, best served by adopting generic (e.g. depression and anxiety) rather than diagnostic‐system‐specific terms and considering the archetypal features of each condition in a general way.

The remainder of the article will be divided up into three sections. In the first, we will introduce a unified model of economic decision making we have developed to help both organize the empirical evidence relating to the putative sources of impairment in different mental disorders and provide a framework for the development of hypotheses regarding the underlying neuropsychological and neurobiological mechanisms. In the second part, we will apply the model to the four disorders – first setting up differential hypotheses about the role of different stages in decision‐making, neuropsychological processes, and neural systems in each disorder, and then selectively surveying the extant empirical evidence in light of these hypotheses. We conclude by identifying issues that merit further investigation.

## An integrated neuroeconomic model of decision making

**Figure 1:**
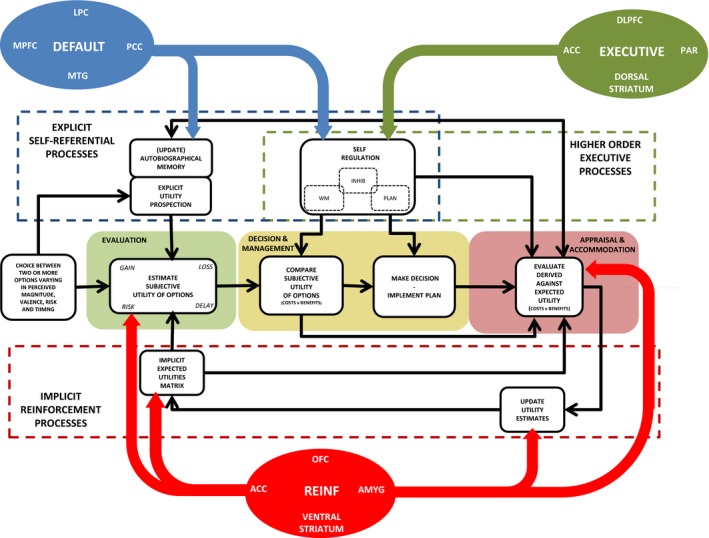
A schematic representation of an integrated neuroeconomic model highlighting the complex interplay between multiple psychological systems and neural circuits in the control of the decision‐making process. The decision‐making process itself is divided into three distinct stages: *Evaluation* – where an estimate of the subjective utility of available choice options is made taking into account memory and learning from prior experience as well as prospection about future value mediated by implicit reinforcement learning mechanisms (encoded in a utility matrix) and explicit self‐referential processes (autobiographical memory); *decision and management* – during which the subjective utility assigned to competing alternatives is compared in terms of overall costs and benefits and a decision plan is implemented – processes heavily influenced by higher order executive functions; *appraisal and accommodation –* utility actually derived from decision is estimated (again on the basis of explicit and implicit value systems) and compared with prior expectations to generate a prediction error signal which drives learning and updates implicit and explicit value estimates for particular experiences and choices as represented by the feedback loops in the figure. The model proposes that these decision‐making stages are primarily controlled by three distinct brain systems: the default mode network (MPFC, medial prefrontal cortex; PCC, posterior cingulate cortex; LPC, lateral parietal cortex; MTG, medial temporal gyrus) primarily implicated in self‐referential cognition but also in some aspects of self‐regulation; executive control system (DLPFC, dorsolateral prefrontal cortex; ACC, anterior cingulate cortex; PAR, parietal cortex) which mediates top‐down control during self‐regulation and planning; and reinforcement evaluation and learning circuits (OFC, orbitofrontal cortex; AMYG, amygdala; ACC, anterior cingulate cortex, REINF, reinforcement)

Figure 1 illustrates the core features of our integrated decision‐making model and highlights the complex interplay underpinning neuropsychological systems (see also Kalueff, Stewart, Song, and Gottesman, 2015). The model has several general characteristics.

### Neuropsychological decision‐making stages

The decision‐making process itself is divided into three distinct stages: (a) evaluation, (b) decision and management, and (c) appraisal and accommodation.

#### Evaluation

This stage involves processes whereby subjective utility estimates are generated for each potential outcome. This involves the integration of information related to parameters, such as valence (win or lose), magnitude (large or small), timing (now or later), and probability (likely or unlikely) – for each possible option. This provides a subjective estimate of the cost/benefit and timing of each outcome. We assume that this is influenced by both the implicit value system of an individual and explicit thought processes. The implicit value system – which we refer to as the *utility matrix –* involves personal preference about content (if an individual prefers apples to oranges, apples will be assigned a higher utility than oranges in the matrix) but also timing (whether a person dislikes risk or delay) of outcomes. Τhe utility matrix is *not* considered a fixed element, but is automatically updated in the light of the evaluation of the consequences of decisions. The explicit value system involves self‐referential autobiographical processes allowing reflection on the experience of prior choices and envisaging potential outcomes. Although the specific weighting of the influences of implicit and explicit processes is not specified in the model, it is assumed that abnormalities in either set of processes could disrupt outcome appraisal or influence evaluation processes in those with mental disorders.

#### Decision and management

This stage involves comparisons of the subjective utility estimates of the available alternatives, the choice of one option over the other, and the implementation of a plan to ensure that the chosen option is enacted effectively. We assume higher order self‐regulatory functions of executive control to be especially important during this phase. In particular, choice between options will involve working memory and inhibition, while goal attainment will involve effective planning, inhibition, and self‐organization.

#### Appraisal and accommodation

This stage is underpinned by reinforcement learning processes – whereby a prediction error is generated through a comparison of expected and derived utility, which then feeds back to both the subjective expected utility matrix and autobiographical memory. The evaluation of the discrepancy between predicted versus derived utility is influenced by the current utility matrix, the self‐regulation processes required to hold intertemporal information in mind, and autobiographical memory concerning prior choices.

### Neurobiological substrates

The model focuses on three distributed and interacting brain systems. These include (a) the neural circuitry controlling self‐referential cognitions – the so‐called default mode network; (b) the network involved in top‐down *executive control* – including lateralized regions of the prefrontal and parietal cortex and the dorsal striatum; and (c) the cortical–subcortical circuits implicated in *reinforcement learning, encoding of value, and emotion processing* – including ventral regions of the prefrontal cortex (such as the orbitofrontal cortex and the anterior cingulate cortex), ventral striatum, insula, and amygdala. These networks interact with each other, providing an additional level of complexity. In the next section, we examine the role of the three core neuropsychological domains implicated in the model.

#### Self‐referential processes

Effective decision making is facilitated if one can disengage from the immediate environment, stand back unencumbered by the influence of imminent and tangible incentives, and consider priorities across an extended timeframe – integrating one's current personal values and past experiences into a coherent picture while envisioning future possibilities. In recent years, there has been a renewed interest in task‐independent self‐referential cognition of this sort (Smallwood & Schooler, 2015) and the ways in which such cognitive processes may be disturbed in mental disorders (Andrews‐Hanna, Smallwood, & Spreng, 2014).

The role of such processes in decision making has recently been discussed (Sonuga‐Barke & Fairchild, 2012). A crucial feature is the idea that individuals construct a coherent autobiographical script about the personal meaning and subjective utility of past choice outcomes and future choice options based on a well‐integrated concept of themselves as effective economic agents (D'Argembeau et al., 2014). This arises out of the ability to reflect on past experiences to provide a basis for future choices (Addis, Wong, & Schacter, 2007). The final self‐referential element in economic decision‐making involves self‐projection to compare future outcome scenarios (Lin & Epstein, 2014) to estimate the subjective value of each choice. In this way, self‐referential processes play a positive role in decision making. However, there is also a potential downside. This arises from the fact that when not properly regulated – for instance, occurring in the wrong setting or at the wrong time or when an individual engages in excessive negative rumination – self‐referential processes can lead to mind‐wandering or daydreaming which disrupts performance (Christoff, Gordon, Smallwood, Smith, & Schooler, 2009) because of lapses of attention (Sonuga‐Barke & Castellanos, 2007).

The neural substrates of self‐referential cognition appear to be located along a cortical midline axis with two major hubs – medial prefrontal cortex (extending ventrally to include dorsal anterior cingulate cortex) and medial parietal cortex – in particular, the posterior cingulate cortex and precuneus (Snyder & Raichle, 2012). These regions, together with circuits involving more lateral elements (i.e. temporal‐parietal junction and medial temporal gyrus), form an interconnected set of regions known as the default mode network. Because of its size, location, and extensive range of connections to cortical and subcortical structures, the cortical midline axis operates as a coordinating hub, bringing together internally generated thoughts and externally available information about oneself and others into a coherent narrative that integrates past experiences to bear on future actions (Moran, Kelley, & Heatherton, 2013). The regions of the default mode network interact with brain regions responsible for cognitive control (Smallwood, Brown, Baird, & Schooler, 2012), reinforcement processing (Cauda et al., 2011), memory (James, Tripathi, Ojemann, Gross, & Drane, 2013), and emotion processing and regulation (Chase, Moses‐Kolko, Zevallos, Wisner, & Phillips, 2014). Functional magnetic resonance imaging (fMRI) studies have shown that default mode nodes form a functional network at rest with temporal coherence between constituent regions which partly overlaps with patterns of white‐matter connectivity (Greicius, Supekar, Menon, & Dougherty, 2009; Vertes & Bullmore, 2015). The activity of the default mode network is attenuated following transitions to goal‐directed tasks requiring effortful, focused attention, and cognitive engagement (Snyder & Raichle, 2012). The centrality of the default mode network in economic decision making is supported by findings from a recent meta‐analysis of brain activations during value computation (Clithero & Rangel, 2014). More specifically, with regard to economic decision‐making, fMRI studies have implicated a medial frontotemporal axis as a putative neural mechanism underpinning the bridge between self‐referential retrospection and prospection (Buckner, Andrews‐Hanna, & Schacter, 2008) with medial prefrontal cortex and medial temporal gyrus interacting to facilitate prospection (Lavallee & Persinger, 2010; Spreng & Grady, 2010). This is consistent with data showing that the latter is involved in autobiographical memory retrieval, which provides the foundation for internal mentation (Lavallee & Persinger, 2010; Tulving, 2002), while medial prefrontal cortex is implicated in self‐related future simulations central to the consideration of future choice outcomes (Kim, 2012) and complex perspective‐taking processes (Van Hoeck et al., 2013). Medial prefrontal–posterior cingulate cortex circuits regulate self‐initiated goal formation and planning in conjunction with dorsal attention networks (Spreng, Stevens, Chamberlain, Gilmore, & Schacter, 2010). Furthermore, these brain regions appear to play a role, in concert with the orbitofrontal cortex, in sustaining long‐term goal states. In addition, the frontopolar cortex plays a complementary role in protecting the execution of long‐term economic plans to allow implementation of decisions (Koechlin & Hyafil, 2007).

Evidence for disruptions of task‐independent and self‐referential thought and the associated default mode network in mental disorders has been available for some time (Broyd et al., 2009). Two general models have been proposed. First, mental disorders often impair self‐referential processes (reducing the ability to form autobiographical memories or envision the future), on the one hand, or distort the content of such processes on the other (leading to a focus on negative experiences or personal failures; Andrews‐Hanna et al. 2014). These may, in turn, impair particular stages of the decision‐making process, disrupt the transition between decision stages, or introduce pathological biases into others. Altered self‐referential processes are reflected in patterns of altered connectivity during rest and introspection either within or between default mode hubs and other systems involved in cognitive control or emotion processing. Second, there is evidence that some individuals with mental disorders show impaired modulation of default mode activity during task performance (Sonuga‐Barke & Castellanos, 2007). This allows intrusive self‐referential thoughts (e.g. mind wandering) to undermine task performance. Such intrusions could have multiple origins, and these are likely to differ between disorders. Intrusions of self‐referential thought during task performance may also be content‐driven – for example, they could be associated with a compulsion to dwell on past events or worry about the future (Servaas, Riese, Ormel, & Aleman, 2014), in both cases leading to excessive default mode activity.

#### Executive control

Executive function is an umbrella term that refers to a heterogeneous grouping of top‐down processes that allow individuals to regulate their thoughts and behavior to successfully engage in purposeful, goal‐directed, and future‐oriented actions (Suchy, 2009). There is general consensus among researchers that EFs comprise three core types: inhibition, working memory, and cognitive flexibility (Lehto & Elorinne, 2003; Miyake et al., 2000). Inhibition encompasses the suppression of prepotent responses (i.e. response inhibition) and control of interference from extraneous stimuli and distracting internal mental representations (Diamond, 2013). Working memory has multiple interrelated components. In the most well‐regarded model, visuospatial and verbal working memory work together with a central executive to allow the simultaneous holding in mind and manipulation of multiple units of information (Baddeley & Hitch, 1974). Finally, cognitive flexibility is the process involved in the changing of perspective and response sets – adjusting how one thinks about something – and being sufficiently flexible to change in response to demands and/or priorities (Diamond, 2013). Additional higher order executive functions, such as reasoning, problem solving, and planning, are conceptualized as processes that build on the aforementioned more basic processes (Collins & Koechlin, 2012; Lunt et al., 2012). Despite continued debate, it is acknowledged that these different executive processes represent separable but moderately correlated constructs (Miyake et al., 2000).

From the perspective of our model, executive functions are related to decision making in several ways. First, in general terms, they provide the basis for deliberative processes and development of decision plans (Bickel, Pitcock, Yi, & Angtuaco, 2009). Second, cognitive flexibility allows one to consider alternative options simultaneously (Diamond, 2013). Third, inhibitory control provides the time to reflect effectively on choice alternatives, while working memory allows multiple units of information to be assimilated and compared (Amso, Haas, McShane, & Badre, 2014). Fourth, executive control is also required for planning the implementation of the selected option (choice), together with the prospective information derived from task‐independent processes (Suchy, 2009). Additionally, executive functions, in particular, inhibitory control, are involved in resisting interference from competing options during the implementation of plans (Sylvester et al., 2003).

Executive functions are most engaged when the novelty or complexity of a situation makes it impossible to rely only on automatic responses or when control is required in the face of motivationally salient events (so called hot executive function settings; Kerr and Zelazo 2004). This latter situation is especially common in the context of economic decision making (Krain, Wilson, Arbuckle, Castellanos, & Milham, 2006). Executive functions are also implicated in timing, which may be important for decision‐making processes – especially perceptual timing and temporal foresight (Noreika, Falter, & Rubia, 2013). Inhibitory control and working memory are pivotal in tasks involving duration discrimination, time reproduction (Noreika et al., 2013), and temporal foresight (Lin & Epstein, 2014).

Traditionally, different executive functions have been localized to specific divisions of the prefrontal cortex: dorsolateral prefrontal cortex for working memory (Fuster, 1999); medial prefrontal cortex (including the anterior cingulate) for flexibility (Bush et al., 1999); ventral prefrontal cortex (including orbitofrontal and ventromedial) for inhibition (Tremblay & Schultz, 2000); and frontal pole (including anterior portions of the dorsolateral prefrontal cortex) for the higher order integration of executive functions (Koechlin, Basso, Pietrini, Panzer, & Grafman, 1999). However, it is now clear that the neural substrate of executive functions is better understood in terms of brain networks implicating basal ganglia, thalamus, cerebellum, and cortical regions outside the prefrontal cortex. For instance, working memory is controlled by a frontoparietal network (Darki & Klingberg, 2015) with the response inhibition network incorporating projections to the basal ganglia (in particular the caudate) and the thalamus (Suchy, 2009). In addition, both the right and left dorsolateral prefrontal areas and the superior medial frontal lobe have been implicated in tasks that involve cognitive switching (Jurado & Rosselli, 2007). Shifting processes also activate parietal lobe and left middle and inferior prefrontal gyri (Jurado & Rosselli, 2007). A meta‐analysis (Houde, Rossi, Lubin, & Joliot, 2010) of task‐based fMRI studies exploring executive functions in youth found that bilateral prefrontal areas, including the dorsolateral prefrontal and inferior prefrontal cortices, extending to the insular cortex, as well as related posterior parietal and occipital areas, were consistently activated in youth, mirroring findings in adults (Niendam et al., 2012).

Executive functions are essential for cognitive, social, and psychological development (Diamond, 2013), as well as positive academic (Borella, Carretti, & Pelegrina, 2010) and professional outcomes (Bailey, 2007). Given this, it comes as no surprise that a growing body of evidence points to executive function impairment in mental disorders, including, among others, substance use disorders (Baler & Volkow, 2006), schizophrenia (Barch, 2005), and obsessive compulsive disorder (Penades et al., 2007). In general terms, there are a number of models of executive function deficits in psychopathology. First, there are models that postulate core executive function deficits with disruptions in top‐down control contributing to the main deficits that characterize the disorder (Barkley, 1997; Oosterlaan, Logan, & Sergeant, 1998). Second, there are models that implicate altered energetic processes as a factor undermining the supply of effortful cognitive control (Sergeant, 2000), concluding that the degree of inhibitory control is dependent on the individual's state and the allocation of energy to the tasks. Third, models propose specific deficits in the regulation of emotionally and motivationally charged responses (Moon & Jeong, 2015). Finally, there are models that argue that executive function deficits arise out of other aspects of a disorder – so for instance in Eysenck et al.'s attentional control theory (Eysenck, Derakshan, Santos, & Calvo, 2007), the worry and rumination that characterize affective disorders limit the resources available for effective executive control.

#### Reinforcement processes‐ valuation and learning

A choice between two or more options is predicated on the ability to make a judgment about the likely subjective utility to be derived from the respective options. Such processes are complex and likely to involve, on the one hand, conscious and explicit autobiographical memory processes, and on the other hand, implicit reinforcement‐related processes. With regard to the latter, it is now clear that specific neural circuits are implicated in (a) the prospective valuation of possible events, (b) the appraisal of outcomes, and (c) the updating of what we have termed the utility matrix that informs these through the process of reinforcement learning. Through this neurobiologically based set of processes, individuals are able to adapt their behavior in complex environments, learn from experience, and predict the likely consequences of their actions (Dayan & Niv, 2008; Stringaris, 2015).

At the core of these reinforcement processes is a mechanism through which the discrepancy between the subjective utility actually derived from a choice compared with that initially predicted is computed. This discrepancy is encoded by a prediction error signal, which is positive if the outcome is better and negative if the outcome is worse than expected (Schultz, Dayan, & Montague, 1997; Stringaris, 2015) – leading to the updating of the individual's estimation of the value of choices (Rushworth, Noonan, Boorman, Walton, & Behrens, 2011; Stringaris, 2015). These processes can be modeled formally using reinforcement learning algorithms, such as the temporal difference learning rule (Sutton & Barto, 1998), and the resulting models can be fitted to fMRI data to investigate neural signals related to expected value or prediction error computations in humans (O'Doherty, Dayan, Friston, Critchley, & Dolan, 2003).

The ability to generate value signals for different options is fundamental to the evaluation phase of decision making. This process involves converting all the anticipated benefits and costs of different choice alternatives into a common ‘currency’ so that they can be compared with each other (Chib, Rangel, Shimojo, & O'Doherty, 2009; Padoa‐Schioppa, 2011; Stringaris, 2015). The decision maker also needs to be able to integrate across a range of different parameters (e.g. potential gains or losses, probability of outcomes, and delay until outcome receipt) when selecting between options. In addition, reinforcement processes are involved in the evaluation stage of decision making in at least two ways. First, they mediate the hedonic experience associated with rewarding outcomes, that is, they provide a neural substrate for the subjective experience of reward. Second, they enable the individual to learn from experience and update the expected value representations that influence future decisions (as described above).

A number of brain regions and networks have been implicated in valuation and learning. There is extensive evidence that the ventromedial prefrontal cortex, orbitofrontal cortex, and ventral striatum are involved in coding the value of options, particularly when a choice is required (Kim, Shimojo, & O'Doherty, 2006; Lebreton, Jorge, Michel, Thirion, & Pessiglione, 2009; Plassmann et al., 2010). Interestingly, such value signals appear to be modulated by parameters which influence choice behavior, such as valence, probability/risk, delay, and the individual's motivational state (e.g. Gottfried, O'Doherty, and Dolan 2003). The ventromedial prefrontal/orbitofrontal cortex is also activated during the receipt of rewarding outcomes, regardless of modality and type of reward (i.e. it is responsive to primary and secondary reinforcement; Kim et al., 2006; Liu, Hairston, Schrier, and Fan 2011). Prediction error signals appear to be encoded by dopaminergic neurons in the striatum (and particularly ventral striatum), the ventral tegmental area, amygdala, and orbitofrontal cortex (D'Ardenne, McClure, Nystrom, & Cohen, 2008; Niv, Edlund, Dayan, & O'Doherty, 2012; O'Doherty et al., 2003). Negative outcomes (both anticipated and received) are processed by specific brain regions such as the amygdala and insula that are known to play a broader role in emotion processing and regulation (Barrett, Mesquita, Ochsner, & Gross, 2007; Ochsner & Gross, 2005). The amygdala, in particular, is heavily connected to other key elements of the reinforcement system (Kim et al., 2011).

Neuroimaging studies investigating the processing of rewards or negative outcomes have demonstrated that the brain is sensitive to the valence of outcomes, with ventromedial prefrontal/orbitofrontal cortex activation increasing when rewards are received and decreasing in response to loss outcomes (Kim et al., 2006; Tom, Fox, Trepel, & Poldrack, 2007). A meta‐analysis of fMRI studies of decision making found that rewarding outcomes (encompassing monetary rewards) activated the striatum, anterior insula, medial orbitofrontal, and rostral anterior cingulate cortex (Liu et al., 2011). Negative outcomes also triggered striatal and anterior insular activity, but additional activations were observed in lateral orbitofrontal cortex, inferior frontal gyrus, dorsal anterior cingulate cortex, and amygdala (Liu et al., 2011). This meta‐analysis suggests that brain regions involved in processing positively and negatively valenced information are highly overlapping, but the direction of the change in activity may vary within the same regions (e.g. medial orbitofrontal cortex or ventral striatum) according to valence (Kim et al., 2006). Interestingly, the direct contrast of rewarding versus negative outcomes revealed multiple regions (e.g. medial orbitofrontal cortex and ventral striatum) that were more sensitive to the former than the latter, whereas only lateral orbitofrontal cortex and caudal regions of the anterior cingulate were more sensitive to negative outcomes.

Functional MRI studies attempting to disaggregate value and risk processing in healthy adults have demonstrated that these parameters are encoded in partially distinct brain networks. While value signals are primarily encoded in orbitofrontal cortex and ventral striatum as noted above, risk or outcome uncertainty is correlated with lateral orbitofrontal cortex and dorsal anterior cingulate activity (Christopoulos, Tobler, Bossaerts, Dolan, & Schultz, 2009; Tobler, O'Doherty, Dolan, & Schultz, 2007). In addition, a region in medial frontal cortex appears to integrate these signals according to the individual's risk attitudes (Tobler et al., 2007). Such findings implicating the lateral orbitofrontal cortex in risk processing are consistent with neuropsychological studies showing heightened risk‐taking following orbitofrontal cortex lesions (Hsu, Bhatt, Adolphs, Tranel, & Camerer, 2005; Sanfey, Hastie, Colvin, & Grafman, 2003) or at least lesions that disrupt adjacent fibers (Rudebeck, Saunders, Prescott, Chau, & Murray, 2013). Finally, a number of studies have investigated neural activity when the individual is selecting between immediate and delayed rewards (Kable & Glimcher, 2007; McClure, Laibson, Loewenstein, & Cohen, 2004). Using an intertemporal choice task, Kable and Glimcher (2007) showed that ventral striatal, medial orbitofrontal cortex, and posterior cingulate cortex activation were inversely related to the length of the delay before reward delivery. Consequently, these regions appear to play an important role in temporal discounting of delayed rewards. However, it should be noted that there are substantial individual differences in rates of temporal discounting, even among healthy adults and children (Olson et al., 2009), and such differences appear to map onto neural activity (e.g. individuals showing the shallowest discounting functions in their choice behavior also exhibited the weakest effects of delay imposition on neural activity; Kable & Glimcher, 2007)] In related work, McClure et al. (2004) observed increased medial orbitofrontal cortex, ventral striatum, and posterior cingulate cortex activity when subjects selected immediate monetary rewards, whereas lateral orbitofrontal cortex and dorsolateral prefrontal cortex were activated during intertemporal choice regardless of delay. Similar results were obtained using primary reinforcement (i.e. immediate or delayed juice delivery; McClure, Ericson, Laibson, Loewenstein, & Cohen, 2007). These results were interpreted as evidence that distinct neural systems (impulsive/automatic vs. deliberative) were in competition during intertemporal choice.

Learning and evaluation processes have been implicated in mental disorders in a number of ways (Luman, Tripp, & Scheres, 2010; Sonuga‐Barke, 2011). First, individuals with mental disorders may show a general insensitivity to reinforcement (both positive and negative), which influences both the encoding of cues and processing of outcomes (e.g. depression – Pizzagalli, 2014). Second, certain forms of psychopathology may be underpinned by deficits in reward or punishment learning, due to impairments in generating stimulus–response–outcome representations (e.g. schizophrenia; Waltz, Frank, Robinson, and Gold 2007). This could be due to insensitivity to either positive or negative feedback and reduced ability to adjust behavior according to environmental contingencies. Third, individuals with mental disorders may display a specific insensitivity to either rewarding or punishing outcomes. Fourth, cue and outcome processing could be essentially intact, but the process of comparing different outcomes may be disrupted (i.e. affecting the decision/implementation phase of decision making). Fifth, individuals with mental disorders might display normal sensitivity to external reinforcement but deficits in intrinsic reinforcement or vice versa. Related to this concept, individuals with psychopathology may show domain‐specific impairments in reinforcement, for example, reduced sensitivity to social reinforcement (praise) but normal sensitivity to monetary reinforcement (Demurie, Roeyers, Baeyens, & Sonuga‐Barke, 2011; Scott‐Van Zeeland, Dapretto, Ghahremani, Poldrack, & Bookheimer, 2010). The final set of models suggests that the preference structures that guide the evaluation process are altered in individuals with psychopathology, perhaps due to early adversity or living in unpredictable environments (e.g. conduct disorder; Sonuga‐Barke, 2014). According to this view, such individuals are capable of evaluating options, implementing decisions, and appraising outcomes, but the weighting of different parameters, such as risk or delay, in the evaluation process is altered in a relatively stable manner.

## Disorder‐specific hypotheses

**Figure 2:**
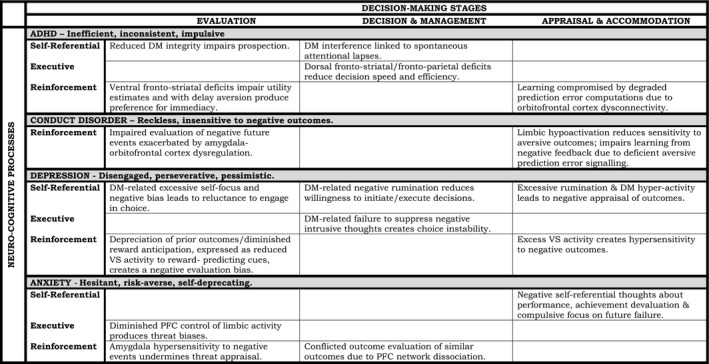
Disorder‐specific hypotheses mapped onto the decision‐making stages outlined in our neuroeconomic model. Key: ADHD: attention‐deficit/hyperactivity disorder; DM: default mode network; PFC: prefrontal cortex; VS: ventral striatum [Correction added on 7 January 2016, after first online publication: The previous incorrect table was published without content and it has now been replaced with the corrected version.]

In this section, we first present individual hypotheses regarding the different behavioral expressions, and associated neurobiological and neuropsychological processes, of impaired decision making in ADHD, CD, anxiety, and depression based on our neuroeconomic model (Figure 1). We acknowledge that additional systems (e.g. autonomic nervous system) are almost certainly affected in these disorders and implicated in decision making, but due to space limitations, these systems are only considered briefly here. Our primary aim is to explore potential differences between disorders in terms of decision making and motivational styles. Our hypotheses, therefore, emphasize *differences* rather than *similarities* between disorders – a point which is particularly relevant when contrasting decision making in the highly overlapping conditions of anxiety and depression. Finally, although a systematic review of evidence is beyond the scope of this article, we briefly consider indicative evidence, focusing on data from children and adolescents where available – although it must be noted that we have frequently had to rely on adult data, with all of the caveats this implies. Figure 2 provides a summary of the hypotheses for the four disorders as these relate to different decision‐making stages and neurocognitive systems.

## Attention‐deficit/hyperactivity disorder

### Background

Attention‐deficit/hyperactivity disorder is a prevalent, debilitating life‐span condition marked by developmentally inappropriate levels of hyperactivity, impulsivity, and/or inattention (Faraone et al., 2015). Clinically, it is highly comorbid with both externalizing (e.g. CD) and internalizing (e.g. depression) problems. Pathophysiologically, it is heterogeneous, with different ADHD individuals displaying marked variation in the profile of contributing factors and deficits across multiple brain networks (Sonuga‐Barke, Bitsakou, & Thompson, 2010). Multimodal treatment is supported empirically, with medication targeting the core and associated symptoms (Banaschewski et al., 2006) and behavioral approaches used to treat co‐occurring problems, such as antisocial behaviors and social skills deficits (Daley et al., 2014).

### Core hypotheses regarding impaired decision making in ADHD

Alterations in multiple brain systems interact to disrupt self‐referential, executive, and reinforcement processes that act across processing stages to produce decision making that is both *deficient* (i.e. inefficient, insufficiently reflective, and inconsistent) and *impulsive* (*biased* toward immediate over delayed alternatives).

#### Evaluation

(a) Disturbed prospection of future events and states due to disrupted connectivity between core midline and lateralized nodes of the default mode network combines with deficient reinforcement signaling within ventral frontostriatal circuits to impair the ability to estimate the subjective utility of choice alternatives. (b) A bias toward choosing immediate overdelayed rewards arises from a combination of reduced signaling of future reinforcement in the ventral striatum and heightened aversion to delay linked to amygdala hyperactivation.

#### Decision and management

(a) A generalized deficit in top‐down executive control mediated by disruptions in frontostriatal and frontoparietal circuits reduces the speed and efficiency of decision making – effects compounded by spontaneous lapses in attention linked to interference from default mode‐related task‐independent thoughts. (b) Impairments in the ability to generate and implement plans consistently without changing or reversing preferences are linked to dysconnectivity between default and executive networks (medial prefrontal and dorsolateral prefrontal cortex) and failures to resist the lure of competing choice alternatives and distracting influences due to executive dysfunction.

#### Appraisal and accommodation

The ability to compare predicted and derived utility (i.e. prediction error signal) and thus learn from experience is degraded by disruptions in anterior cingulate cortex–orbitofrontal cortex connectivity.

### Empirical indications

#### Dysregulation of self‐referential processes

To date, no direct study of self‐referential processes in decision making in ADHD has been undertaken. However, evidence linking ADHD to dysfunction within the default mode network, hypothesized to subserve effective self‐referential cognition, has accumulated in recent years. Resting state fMRI studies have demonstrated disrupted connectivity between midline hubs of the default mode in ADHD (Castellanos et al., 2008). A recent study found a general developmental lag in intrinsic default mode network structure in ADHD and disrupted connections to executive and attentional networks (Sripada, Kessler, & Angstadt, 2014). Other studies found that ADHD individuals have difficulties in regulating default mode activity appropriately to respond to external demands, leading to excessive activity within this network during task performance (Helps et al., 2010; Liddle et al., 2011). In terms of evidence relating to the dysregulation of putative self‐referential processes underpinned by default mode dysfunction in ADHD, there is both direct and circumstantial evidence. Most directly, Seli, Smallwood, Cheyne, & Smilek (2015 recently reported evidence of a link between ADHD in young adults and the sort of spontaneous, dysfunctional, and uncontrolled mind‐wandering associated with disorganized introspection and lapses of attention during external tasks – contrasting this with the deliberate, well‐regulated and functional self‐referential thought required for effective prospection and planning. Direct evidence linking this form of maladaptive mind‐wandering and attention in ADHD is not yet available although mind‐wandering has been directly linked to attentional lapses (Smallwood, McSpadden, Luus, & Schooler, 2008) and low‐frequency signatures in reaction time data, characteristic of such lapses, have been observed repeatedly in ADHD (Karalunas, Geurts, Konrad, Bender, & Nigg, 2014). More circumstantially, deficits in autobiographical and prospective memory have been observed. Fuermaier et al. (2013) demonstrated ADHD‐related deficits in both self‐rated and objectively assessed prospective memory. Furthermore, the same group narrowed this effect down specifically to deficits in long‐term planning rather than the recall of planned action, plan integrity, or self‐initiation. Support for difficulties in long‐term planning functions also comes from a number of sources including in relation to goal setting (Nyman et al., 2010), ‘if‐then’ plans (Gawrilow, Merkt, Goossens‐Merkt, Bodenburg, & Wendt, 2011) and planning scripts (Desjardins, Scherzer, Braun, Godbout, & Poissant, 2010). Prospective memory, especially time‐, as opposed to event‐based memory, also appears to be impaired in ADHD (Talbot & Kerns, 2014). Fabio & Capri (2015) found that deficits in autobiographical memory inhibited the ability of ADHD children to access personal events in the past while (Klein, Gangi, & Lax, 2011) demonstrated an association between ADHD and disorganized personal narratives when asked to access episodic self‐referential terms. Scholtens, Rydell, & Yang‐Wallentin (2013) found reduced future orientation regarding academic matters in adolescent ADHD.

#### Impaired executive control

There is now considerable evidence relating to executive functions in ADHD. Meta‐analyses provide evidence of ADHD‐related deficits in inhibition (Lipszyc & Schachar, 2010), interference control (Lansbergen, Kenemans, & van Engeland, 2007), and working memory (Alderson, Kasper, Hudec, & Patros, 2013), which are likely to impact on decision making. Deficits in higher order executive processes such as attentional flexibility and short‐term planning are also apparent (Willcutt, Doyle, Nigg, Faraone, & Pennington, 2005). This evidence from neuropsychological tests converges with neuroimaging evidence, which highlights ADHD‐related alterations in lateralized frontoparietal and frontostriatal structure (Pironti et al., 2014) and function (Cortese et al., 2012). The relationship between executive control and decision making in ADHD has been examined in two ways. First, there is a large and growing literature on decision making about rewards under conditions of uncertainty and risk – so‐called hot executive settings. Results to date are mixed and open to interpretation (Groen, Gaastra, Lewis‐Evans, & Tucha, 2013). Around 50% of these studies found that ADHD individuals make poorer and riskier decisions than controls. However, because of the complex and cognitively demanding nature of the tasks and their reliance on multiple processes, these positive results are challenging to map onto specific cognitive processes (Brand, Franke‐Sievert, Jacoby, Markowitsch, & Tuschen‐Caffier, 2007). Probably most informative is the Cambridge Gambling Task which allows different decision‐making elements to be disentangled (Manes et al., 2002). The studies using this task found that ADHD individuals make suboptimal choices, but this relates to problems in processing and adjusting information about risk or to delay aversion (i.e. choosing the earliest available option; see below) rather than risk proneness per se (Coghill, Seth, & Matthews, 2014; DeVito et al., 2008). Second, a number of studies have examined the links between decision making and executive functions in ADHD by including paradigms measuring both processes. For instance, Duarte, Woods, Rooney, Atkinson, & Grant (2012) found that suboptimal decision making on the Iowa Gambling Task was related to working memory deficits in ADHD. Drechsler, Rizzo, & Steinhausen (2008) found that poor decision making on a gambling task was related to inhibitory control deficits, but not working memory problems, in ADHD. However, no studies have specifically explored the neural basis of the cognitive impairments leading to poor decision making in ADHD.

#### Impaired reinforcement processes

Imaging studies have implicated structural alterations in the neural circuits and regions that mediate reinforcement‐related processes – including the orbitofrontal cortex (Hesslinger et al., 2002) and the ventral striatum (Carmona et al., 2009). In terms of difficulties with processing signals of future reinforcement, a recent meta‐analysis confirmed ventral striatal hyporesponsiveness in individuals with ADHD (Plichta & Scheres, 2014), which appears independent from dysfunction in executive control networks (Carmona et al., 2012). Wilbertz et al. (2012) also found reduced orbitofrontal cortex sensitivity to reward magnitude changes in adult ADHD. There is evidence of functional hyperconnectivity between core hubs of the reward circuit (Tomasi & Volkow, 2012). Evidence from behavioral tasks gives a rather mixed picture (Luman, Sergeant, Knol, & Oosterlaan, 2010), with some studies suggesting oversensitivity to rewards (Fosco, Hawk, Rosch, & Bubnik, 2015) and others showing hyposensitivity (van Meel, Heslenfeld, Oosterlaan, Luman, & Sergeant, 2011) related to impaired reward‐related prediction error signals (Thoma, Edel, Suchan, & Bellebaum, 2015). In line with our predictions, the most consistent finding relates to an accentuated sensitivity to delay prior to the delivery of reinforcement, which holds regardless of the paradigm used (Yu, Sonuga‐Barke, & Liu, 2015) and seems to reflect a combination of a drive toward immediate reinforcement (Marco et al., 2009), heightened discounting of delayed reinforcement (Scheres, Tontsch, & Thoeny, 2013) and aversion to delay – a desire to escape the negative affect induced by delay (Lemiere et al., 2012). The limited neuroimaging evidence available is consistent with this picture, with increased discounting in ADHD associated with atypical connectivity between the ventral striatum and executive control regions (Costa Dias et al., 2013), while cues of impending delay lead to enhanced activation of limbic regions known to encode aversive stimuli (i.e. amygdala and anterior insula) in ADHD individuals (Wilbertz et al., 2013). Surprisingly, few studies have investigated reinforcement learning per se in ADHD, given the centrality of this process to two highly influential models of ADHD (Sagvolden, Johansen, Aase, & Russell, 2005; Tripp & Wickens, 2008). Two recent studies are particularly relevant here. Luman, Goos, & Oosterlaan (2015) found that children with ADHD learned at the same rate as controls on an instrumental learning task. In contrast, Hauser et al. (2014) observed reduced reward medial prefrontal cortex prediction error signals in adolescents with ADHD during reversal learning task performance, supporting the hypothesis that reinforcement learning is disrupted in ADHD.

### Summary of ADHD‐related research priorities

Perhaps in contrast to other disorders reviewed here, there is already a substantial and growing body of evidence either directly examining key aspects of decision making in ADHD or at least exploring systems and processes hypothesized to be involved in decision making. Much of this work was done in children and adolescents. However, the extant literature lacks integration and the field remains fragmented. Furthermore, key questions remain unaddressed. We feel that three key research priorities are (a) to study the way that explicit self‐referential cognitive and implicit reinforcement‐related processes interact during the evaluation stage of decision making and whether this contributes to the way value is assigned by individuals with ADHD; (b) to explicitly examine the role played by prospection and its neural substrates in decision making about future rewards in ADHD; and (c) to better understand how aberrant reinforcement processing in ADHD influences learning and how this in turn feeds back to affect stimulus evaluation.

## Conduct disorder

### Background

Conduct disorder is characterized by a persistent and pervasive pattern of antisocial and aggressive behavior (American Psychiatric Association, 2013). It typically emerges in either childhood or adolescence and is predictive of antisocial personality disorder (Copeland, Shanahan, Costello, & Angold, 2009; Lahey, Loeber, Burke, & Applegate, 2005; Robins, 1978) and other negative adult outcomes (Burke, Rowe, & Boylan, 2014; Copeland et al., 2009; Moffitt, Caspi, Harrington, & Milne, 2002; Odgers et al., 2007). Although comorbidity rates are highest with other externalizing disorders (e.g. ADHD), there is also substantial overlap with *internalizing* disorders (e.g. depression and anxiety; Fergusson, Horwood, & Ridder, 2005; Lahey & Waldman, 2012). Conduct disorder can be subclassified based on age‐of‐onset (childhood vs. adolescence‐onset; American Psychiatric Association, 2013) or in terms of associated personality characteristics (e.g. callous‐unemotional traits; Burt, Donnellan, Iacono, & McGue, 2011; Frick, Ray, Thornton, & Kahn, 2014). Disruptions in brain regions such as the amygdala and anterior insula that are involved in the processing of valence and emotional and motivational salience are implicated in CD. Structural and functional changes in frontal and temporal regions have also been reported (Baker, Clanton, Rogers, & De Brito, 2015; Fairchild et al., 2011, 2013, 2015; Hyatt, Haney‐Caron, & Stevens, 2012; Rubia, 2011). Treatment options include parenting interventions and multisystemic therapy (NICE 2013). However, atypical antipsychotic medications may be used with patients who are unresponsive to psychosocial interventions (Reyes, Buitelaar, Toren, Augustyns, & Eerdekens, 2006).

### Core hypotheses regarding impaired decision making in CD

Disturbances in reinforcement mechanisms, and related brain circuits, impact evaluation and appraisal/accommodation stages of decision making with specific effects on the processing of negative stimuli, producing *reckless* choices and *insensitivity to negative consequences*.

#### Evaluation

(a) Altered structure and function within, and disrupted connectivity between, amygdala/insula and orbitofrontal cortex generally impair the subjective estimation of negative future events. This reduces the impact of signals of future punishment, risk/uncertainty, and delay on decision making, which is especially pronounced for options combining multiple negative elements (e.g. delayed negative outcomes/distal punishments).

#### Decision and management

We predict that these processes are largely unaffected in CD when not comorbid with ADHD.

#### Appraisal and accommodation

Individuals with CD display normal or enhanced sensitivity to positive or rewarding outcomes but reduced sensitivity to aversive outcomes, blunting their response to, and reducing their ability to learn from, negative feedback. These effects are mediated by hypoactivation of the brain's punishment centers – amygdala and anterior insula – and associated striatal regions, and deficient prediction error signals for aversive events.

### Empirical indications

#### Evaluation of aversive or risk‐related cues

The decision making of children and adolescents with CD or oppositional defiant disorder (ODD) has been studied using four types of tasks involving (a) decision making under risk (outcome probabilities are explicitly presented); (b) decision making under uncertainty (key information is unavailable or where learning is required); (c) reversal learning (where contingencies change); and (d) passive avoidance learning. An early study found no group differences in Iowa Gambling Task (IGT) performance at baseline (Ernst et al., 2003) although unlike controls, CD individuals failed to show performance improvements a week later. More recently, Schutter, van Bokhoven, Vanderschuren, Lochman, and Matthys (2011) found that adolescents with CD/ODD and substance use disorders failed to learn to avoid risky decks associated with large penalties. A study using a modified gambling task obtained similar findings in children with ODD (Luman et al., 2010). In contrast, Fairchild et al. (2009) found that CD was associated with increased risky decision making under risk, suggested heightened sensitivity to gains or reduced sensitivity to losses during the evaluation phase of decision making. Crowley, Raymond, Mikulich‐Gilbertson, Thompson, & Lejuez (2006) found that adolescents with CD and substance use disorders made more risky choices than control subjects using the Balloon Analogue Risk Task (BART). Using the same task, Humphreys and Lee (2011) found that children with comorbid ODD+ADHD made riskier choices than controls, whereas the ODD‐only group was less sensitive to punishment than controls. Collectively, these findings suggest that individuals with CD or ODD have difficulties in adjusting their behavior following negative reinforcement or punishment, whereas studies assessing decision making under risk indicate that CD is associated with altered sensitivity to gains and/or losses during choice evaluation. A recent study investigating intertemporal choice showed heightened temporal discounting in adolescents with CD relative to controls (White, Clanton et al., 2014). Interestingly, these findings remained significant when excluding participants with comorbid ADHD. This suggests a more present‐orientated motivational style in CD or alternatively that CD is independently associated with delay aversion. An fMRI study observed hypoactivation in multiple brain regions in individuals with both CD and substance use disorders (SUDs) during the evaluation phase of decision making (Crowley et al., 2010). The regions implicated included multiple areas in prefrontal cortex, anterior cingulate cortex, insula, and amygdala, as well as temporal and parietal cortices. The CD+SUDs group also showed reduced activation when receiving rewarding feedback in anterior cingulate and temporal and visual cortices, but increased responses to losses in several frontal and temporal regions relative to controls.

#### Impaired reinforcement processes

Learning from aversive events is impaired in those with CD, and such effects are correlated with variations in the severity or persistence of CD. Adolescents with CD, like adults with antisocial personality disorder (Flor et al., 2002), show deficient autonomic conditioning (Fairchild, Stobbe, van Goozen, Calder, & Goodyer, 2010; Fairchild, van Goozen, Stollery, & Goodyer, 2008). Reduced autonomic conditioning at age 3 predicted increased criminal behavior in adulthood (Gao, Raine, Venables, Dawson, & Mednick, 2010), whereas intact conditioning in midadolescence was associated with better outcomes in a high‐risk group (Brennan et al., 1997). Deficient acquisition of conditioning was associated with higher rates of offending within a group of young offenders (Syngelaki, Fairchild, Moore, Savage, & van Goozen, 2013b). Importantly, most studies have not found CD‐related effects on general autonomic reactivity to aversive unconditioned stimuli. While these findings appear to challenge the idea of a general impairment in the processing of negative stimuli, other studies have observed CD‐related reductions in eye‐blink startle or skin conductance responses to aversive stimuli (Fairchild et al., 2008; van Goozen, Snoek, Matthys, van Rossum, & van Engeland, 2004; Syngelaki, Fairchild, Moore, Savage, & van Goozen, 2013a). Consequently, it is currently unclear whether there is a primary deficit in responsiveness to aversive stimuli or a disproportionate impairment in learning from punishment. Indeed, it is plausible that both processes are impaired and reduced sensitivity to aversive stimuli contributes to associative learning difficulties.

Structural MRI studies have observed reduced anterior insula, orbitofrontal cortex, and striatal gray‐matter volume in CD (Fairchild et al., 2011, 2013; Sterzer, Stadler, Poustka, & Kleinschmidt, 2007), suggesting that CD is associated with structural, as well as functional, abnormalities in key regions of the valuation network. Rubia et al. (2009) observed reduced orbitofrontal cortex responses to rewarding outcomes in boys with childhood‐onset CD. A recent study found no group differences in neural activity during reward or loss anticipation when comparing adolescents with persisting and desisting conduct problems and controls (Cohn et al., 2014). However, the persistent disruptive behavior disorder (DBD) group demonstrated reduced ventral striatal activity during reward receipt and increased amygdala responses to receipt of losses. In a passive avoidance task with monetary rewards and punishments, adolescents with DBDs showed weaker expected value signals in ventromedial prefrontal cortex when choosing to respond to stimuli and weaker expected value signals in insula when choosing not to respond (White et al., 2013). They also displayed reduced positive and increased negative prediction error signals in the caudate when receiving feedback. In a follow‐up study using environmental reinforcers, adolescents with DBDs showed reduced expected value signals in caudate nucleus, thalamus, and posterior cingulate cortex when making suboptimal decisions (White, Fowler et al., 2014). These studies support the hypothesis that individuals with CD show deficits in expected value signals for aversive outcomes and altered prediction error signals, although it is currently unclear whether both reward‐related and aversive expected value signals are disrupted.

Functional magnetic resonance imaging studies of emotion processing have demonstrated that adolescents with CD show reduced activity in the dorsal anterior cingulate cortex, amygdala, insula, dorsolateral prefrontal cortex, and caudate nucleus (Fairchild et al., 2014; Lockwood et al., 2013; Passamonti et al., 2010; Sterzer, Stadler, Krebs, Kleinschmidt, & Poustka, 2005). However, in contrast with the findings described above, a study combining psychophysiological and fMRI methods found no significant differences in autonomic fear conditioning between persistent and desisting DBD groups and controls, but increased anterior cingulate cortex/insula responses to the conditioned stimulus in both DBD groups relative to controls (Cohn et al., 2013). These divergent findings may have been explained by elevated anxiety in both the DBD groups.

### Summary of CD‐related research priorities

Three key priorities for future neuroeconomic studies of CD are (a) to investigate systematically decision making using tasks that allow disaggregation of the decision‐making stages identified in this review – even though we hypothesize that decision and management‐related processes are essentially intact in CD, very few studies have examined the intervening processes between evaluation and outcome appraisal. It will also be critical to study the impact of alterations in reinforcement processes (e.g. aversive prediction error signals) on the subsequent evaluation of options and reinforcement learning with both appetitive and aversive stimuli; (b) to examine decision making in social contexts, in order to understand how the decision making of individuals with CD is affected by the presence of peers, and whether their antisocial behavior is related to stable changes in social preferences (e.g. reduced inequity aversion when it concerns others, but heightened sensitivity to (perceived) unfair treatment by others); (c) to investigate the impact of environmental adversity (e.g. being raised in poverty, effects of socioeconomic status gradients, and biological embedding of early‐life stress) on the preference structures that guide the evaluation process, given the strong association between childhood maltreatment, low socioeconomic status, and CD (Caspi et al., 2002; Piotrowska, Stride, Croft, & Rowe, 2015).

## Depression

### Background

Depression is estimated to affect up to 10% of youth by age 16 (Costello, Mustillo, Erkanli, Keeler, & Angold, 2003). Rates increase during adolescence (Thapar, Collishaw, Pine, & Thapar, 2012) and from puberty onwards, and the disorder becomes twice as common in females compared with males (Lewinsohn, Rohde, & Seeley, 1998). Depression is a major contributor to global disease burden (Collins et al., 2011). The presence of co‐occurring disorders is high, with anxiety and DBDs being the most common (e.g. CD; Stringaris, Lewis, & Maughan, 2014). The pathophysiology of depression involves alterations in medial prefrontal networks (anterior cingulate cortex, ventromedial, and orbitofrontal cortex) and related subcortical regions (e.g. amygdala and ventral striatum) which disrupt the processing of, and regulation of responses to, affective and motivationally salient stimuli and events (Kerestes, Davey, Stephanou, Whittle, & Harrison, 2014). Evidence‐based treatments for depression include cognitive behavioral therapy and medication (Maughan, Collishaw, & Stringaris, 2013; Thapar et al., 2012).

### Core hypotheses relating to impaired decision making in depression

Alterations in self‐referential, executive, and reinforcement processes, and their underlying brain networks, interact to produce *disengaged, perseverative*, and *pessimistic* decision making.

#### Evaluation

A dysfunctional attributional style due to negative perceptual biases of past events is compounded by default mode network‐related excessive self‐focusing, which manifests as reluctance to engage in choice behavior. Anticipation of reward is diminished (reflected in decreased ventral striatal activity) which, combined with blunted affective forecasting, exaggerates negative and underestimate positive characteristics of future choice options, contributing to disengaged decisions.

#### Decision and management

Excessive negative rumination on past events reduces the individual's willingness not only to initiate but also to execute future decisions. Failure to suppress default mode network‐mediated negative intrusive thoughts during decision management increases choice instability and the tendency to ineffectively reevaluate ongoing decisions.

#### Appraisal and accommodation

Hypersensitivity to negative outcomes, reflected by increased ventral striatal responses to punishment, coupled with negative appraisal of decisions due to excessive rumination, further contributes to a pessimistic decision‐making style.

### Empirical indications

Models about the influence of emotions on decision‐making stretch back a long time (Loewenstein, Weber, Hsee, & Welch, 2001). Surprisingly, they have received very little empirical testing in the context of psychiatric disorders and, specifically, of depression.

#### Dysfunctional attributional style

Attributional style, the way in which a person explains the causes of positive and/or negative events in their lives, is often altered in depression. As in adulthood (Sweeney, Anderson, & Bailey, 1986), childhood depression is associated with an *internalized* (i.e. self‐blaming), *stable* (i.e. trait like), and *global* (i.e. generalizing across situations) attributional style. Positive events are attributed to *external*,* unstable,* and *specific* causes (Gladstone & Kaslow, 1995). A task‐based fMRI study in adults (Seidel et al., 2012) assessing attributions to social events found that the left temporal pole, the left dorsomedial, and the right ventrolateral prefrontal cortex were significantly more activated in controls versus depressed patients for ‘non–self‐serving’ attributions and in patients versus controls for ‘self‐serving’ attributions. Since, in controls, ‘non–self‐serving’ and, in depressed patients, ‘self‐serving’ attributions are in conflict with the prevailing expected style, the study suggests that higher degree of cognitive control is required to inhibit the prepotent tendency toward either self‐serving or non–self‐serving responses.

#### Excessive rumination and obsessive self‐focus

Rumination can be a maladaptive thinking style as a response to negative mood states, including depression, irritability, and anxiety. It is common in patients with both anxiety and depressive disorders and may contribute to impairment over and above the presence of other psychopathology (McLaughlin & Nolen‐Hoeksema, 2011). Meta‐analytic studies provide compelling evidence for negative rumination in adolescent depression (Rood, Roelofs, Bogels, Nolen‐Hoeksema, & Schouten, 2009). Ruminative thinking can lead to interpretation bias so that ambiguous information is interpreted consistently with the content of ruminations (Mor, Hertel, Ngo, Shachar, & Redak, 2014). In dysphoric individuals, rumination mediates difficulties with making decisions; moreover, it reduces confidence in decisions (van Randenborgh, de Jong‐Meyer, & Huffmeier, 2010b). Rumination can, therefore, bias the evaluation of prospective situations but also the execution of the decision and the interpretation of previous events (appraisal of decisions, see further below). Increased obsessive self‐focused cognition*,* typically associated with negatively distorted autobiographical memories, is common in both adults (for a review, see Ingram, 1990) and in youth with depression. For example, Black and Possel (2013) found that maladaptive self‐referential processing and excessive rumination at baseline predicted increased depressive symptoms 6 months later (Black & Possel, 2013). Additionally, negative memory biases on the self‐referent encoding task at age 6 predicted increased depressive symptoms at age 9 (Goldstein, Hayden, & Klein, 2014). Indeed, compared with healthy controls, individuals with dysphoria experience their decisions as more difficult and had less confidence in their choices and this difficulty was mediated via excessive self‐focused thinking (van Randenborgh et al., 2010b). The same is true for depressed individuals (van Randenborgh, de Jong‐Meyer, & Huffmeier, 2010a). Furthermore, excessive rumination predicts indecision in adults, and this effect is independent of the severity of depression (Di Schiena, Luminet, Chang, & Philippot, 2013). A number of studies have found alterations in functional connectivity in depression as well as in the interplay between the default mode network and other systems, relating such alterations to excessive self‐referential and rumination processes (Marchetti, Koster, Sonuga‐Barke, & De Raedt, 2012; Pannekoek et al., 2014). Rumination has been shown to be correlated with decreased fractional anisotropy (a proxy of white‐matter structural connectivity) in the superior longitudinal fasciculus, the major tract connecting frontal/parietal circuits with the limbic system (Zuo et al., 2012). A seminal study (Sheline et al., 2009) reported increased self‐referential processing related to a failure to deactivate core default mode regions (including ventromedial prefrontal cortex, anterior cingulate, lateral parietal cortex, and lateral temporal cortex) while participants were examining and reappraising pictures. A recent meta‐analysis found increased connectivity between the default mode network and subgenual prefrontal cortex, suggesting that coactivation of these regions is related to behavioral withdrawal and a self‐focused, negatively valenced and withdrawn ruminative state (Hamilton, Farmer, Fogelman, & Gotlib, 2015).

#### Affective forecasting

When one envisions the future, one does not only assess the likelihood of future events but also forms projections for how those events will feel. This is termed affective forecast. Relative to controls, individuals with dysphoria present with blunted affective forecast, expecting future positive events to feel less positive even if they occur (Marroquin & Nolen‐Hoeksema, 2015). This might further strengthen the tendency to avoid future decisions.

#### Reward hyposensitivity

The reward network is altered in adolescents with depressive disorder (Forbes & Dahl, 2012; Kerestes et al., 2014b; Romens et al., 2015) and also in unaffected first‐degree relatives of patients with depression (Olino et al., 2014). One of the most prominent findings is that of reduced anticipation for reward. Recent neuroimaging results demonstrate that activity in the ventral striatum is reduced in adolescent participants with subthreshold and clinical depression relative to healthy comparison subjects during anticipation of monetary rewards (Stringaris et al., 2015). Moreover, diminished ventral striatal response to reward anticipation is linked with anhedonia, rather than low mood, and predicts new onset of depression 2 years later. The reduced response to reward anticipation may underlie a variety of motivational deficits in depression that clinicians traditionally subsume under the construct of *anhedonia*. Importantly, no brain alterations have been found during positive monetary outcomes in depressed subjects compared with controls, strengthening the notion that anticipatory rather than consummatory processes are aberrant in depression (Treadway & Zald, 2011). As such, this process may be particularly relevant in the *evaluation* stage.

#### Hypersensitivity to negative outcomes

Complementing the hyporesponsivity to positive reward, depressed patients also show a hypersensitive to punishment (Kessel, Kujawa, Hajcak, & Klein, 2015) or negative feedback (Eshel & Roiser, 2010). In a recent fMRI study, it was shown that that adolescent with anhedonia, but not those with low mood, showed increased activation in the ventral striatum during negative outcome (Stringaris et al., 2015b). This is in accordance with previous results from adults with anhedonia (Padrao, Mallorqui, Cucurell, Marco‐Pallares, & Rodriguez‐Fornells, 2013). Along with the effect of rumination, hypersensitivity to negative outcome may bias the *appraisal* stage, leading to pessimistic assessment of previous choices.

### Summary of depression‐related research priorities

The following lines of investigation are particularly relevant in relation to the hypotheses discussed above: (a) there is a specific need to investigate the brain correlates of decision‐making processes in depression from a developmental perspective – to address the lack of studies in childhood and adolescence. While the neurobiological underpinnings of the relationship between depression and decision making are being elucidated in adults, there are several unexplored key areas in relation to young people's decision making. For instance, the neuronal correlates of attributional processes have not been specifically and systematically studied in depressed youth. (b) The association between laboratory measures of decision making and more ecologically indices, especially in relation to the evaluation stage need to be established. (c) Finally, we need more experimental studies comparing the effects of cognitive versus behavioral interventions effects on reward‐related decision processes and their brain correlates.

## Anxiety

### Background

Anxiety disorders are common in youth with a prevalence ranging between 5% and 10% (Pine & Klein, 2010) and are probably best understood as extreme expressions of continuously distributed traits (Plomin, Haworth, & Davis, 2009). Anxiety disorders include phenomena that occur in early childhood, such as separation anxiety disorder, and conditions that mainly emerge from adolescence onwards, such as social phobia and panic disorder. There is evidence both for the common etiological underpinnings of these disorders (Rutter, 2011), as well as for the value in distinguishing between them (Pine, 2011). The pathophysiology of anxiety involves alterations in a conserved ‘threat network’ involving subcortical structures, such as the amygdala, that are critical for the acquisition of fear responses (LeDoux, 2000), and frontal areas involved in emotion regulation (Stringaris, 2015). Cognitively, anxious people are more likely to show increased vigilance to threat, characterized by a negativity bias (Bar‐Haim, Lamy, Pergamin, Bakermans‐Kranenburg, & van IJzendoorn, 2007), which is related to amygdala hyperresponsivity to threat (Monk et al., 2008). Medication treatment with selective serotonin reuptake inhibitors is effective in reducing anxiety symptoms, and cognitive behavioral therapy is also effective (James, James, Cowdrey, Soler, & Choke, 2013).

### Core hypotheses relating to impaired decision making in anxiety

Stress induced by heightened levels of performance anxiety arising from *self‐doubt*, combined with hypervigilance for threat, creates a *hesitant*,* risk‐averse,* and *self‐deprecating* decision‐making style.

#### Evaluation stage

(a) Amygdala overactivation, combined with diminished top‐down control in executive circuits (e.g. ventrolateral prefrontal cortex), underpins automatic attentional bias toward threat and leads to the overestimation of negative characteristics of neutral outcomes, especially where those outcomes are ambiguous or uncertain; this in turn leads to excessive risk aversion. (b) Reduced reward valuation during episodes of stress‐induced‐performance anxiety diminishes expected subjective value estimates and decision‐making confidence and is reflected in lower activity in the ventral striatum and ventromedial prefrontal cortex.

#### Decision and management

Dissociation between ventral and dorsal frontal regions gives rise to conflicting positive evaluations alongside anxiety when subjects are faced with ambiguous choices (i.e. when competing outcomes are close in subjective value).

#### Appraisal and accommodation

Anxious self‐referential thoughts about performance, reflected in increased default mode activity during task execution, lead to the devaluation of achieved outcomes – shifting attention away from the present situation toward past or future negative events (compulsive prospection). This leads to impairments in the individual's ability to predict outcomes.

### Empirical indications

#### Attention to threat

Information processing in people with anxiety is biased toward the negative. Anxious children selectively attend to negative information, are distracted by it, and find it difficult to disengage from it (Daleiden & Vasey, 1997). They are more likely to interpret ambiguous information as threatening, in ways that cut across different anxiety disorders and are similar in adults and children (Bar‐Haim et al., 2007), although a reversal of this effect (with bias away from the negative) has been found when individuals are under significant threat (Bar‐Haim et al., 2010). Biases exist for both consciously processed and subliminally presented stimuli. Two stages of biased information processing in anxiety have been identified – an early, fast and automatic or amygdala‐based primary pathway and a secondary, slower system that incorporates contextual information relying on prefrontal processing (Beck & Clark, 1997; Cisler & Koster, 2010; Mogg & Bradley, 1998). Young people with generalized anxiety disorder show excess amygdala activity when briefly presented with angry faces (Monk et al., 2008).

#### Executive control

Anxiety is associated with a reduced ability to recruit executive processes to moderate emotional responses through mechanisms such as attention reallocation or reinterpretation (Pine, 2007; Posne & Rothbart, 2000). From a neural perspective, such regulation happens through cross‐talk between the amygdala and parts of the prefrontal and orbitofrontal cortex: regions are active during emotion regulation such as when appraising emotionally laden situation (Ochsner, Silvers, & Buhle, 2012). It has been demonstrated that increased activity in prefrontal and orbitofrontal areas correlates with reductions in amygdala activity (Banks, Eddy, Angstadt, Nathan, & Phan, 2007; Goldin, McRae, Ramel, & Gross, 2008). In this regard, there is fMRI evidence to support the theory of Eysenck et al. (2007) that anxiety disrupts executive processes, such as inhibition, thus impairing an individual's attentional control over the processing of emotionally salient stimuli. In a study by Monk et al. (2008), hyperactivation in the amygdala shows negative connectivity between this region and the ventrolateral prefrontal cortex suggesting a decreased top‐down control of automatic processes. Moreover, reduced DLPFC activations is present in patients with anxiety but not healthy volunteers during error processing (Fitzgerald et al., 2013).

#### Reward processing and learning

Neuropsychological experiments suggest that anxiety‐related threat perception may bias individuals toward overestimating potential losses (Clark et al., 2012). The presence of anxiety also appears to diminish the expected positive value of outcomes. In healthy volunteers, anxiety is associated with reduced activity in the medial prefrontal cortex following feedback related to both monetary gains and monetary losses (Treadway, Buckholtz, & Zald, 2013). Indeed, anticipatory anxiety diminishes activity in the ventral striatum and medial prefrontal cortex during when these were asked to estimate the subjective value of events and predict outcomes (Engelmann, Meyer, Fehr, & Ruff, 2015). Consistent with animal models of stress activity in the anterior insula, an area known to preferentially encode negative value was increased and the connectivity between medial prefrontal cortex and striatum was diminished during anticipatory stress (Dias‐Ferreira et al., 2009). How these findings about incidental anxiety apply to those with persistent anxiety, typical of most anxiety disorders, remain understudied. Recent results suggest that social reward responsivity to reward valuation in patients with social anxiety disorder may be reduced as reflected by diminished activity in putamen and reduced ventral striatal‐anterior cingulate cortex connectivity (Cremers, Veer, Spinhoven, Rombouts, & Roelofs, 2014). However, these effects may be specific to social reward cues and not apply to monetary rewards (Maresh, Allen, & Coan, 2014). Contingency learning in anxiety has received little attention from researchers. There is some evidence that high‐trait anxious individuals show deficits in the ability to adapt their decisions in the face of aversive stimuli especially when environments became volatile (Browning, Behrens, Jocham, O'Reilly, & Bishop, 2015). This deficit could result in anxious people perceiving aversive events to be less predictable and thus harder to avoid. Future experiments need to test whether other emotional disorders, such as depression, suffer from similar problems.

#### Avoidance

While procrastination is not coterminus with anxiety, both appear related to the avoidance of stressful situations. Hence, anxious behavior is characterized – indeed for some disorders defined by – the avoidance of certain decisions or tasks (American Psychiatric Association, 2013). It appears that the degree of approach or withdrawal from a situation depends on activity in the prefrontal‐striatal‐insular network. Increased prefrontal activity seems to be associated to less approach behavior (Aupperle, Melrose, Francisco, Paulus, & Stein, 2015). Avoidance of decision making may even be present for anxious individuals in so called win–win situations. It appears that dorsal prefrontal areas are associated with anxiety to choice conflict between positive outcomes and predicts the reversal of a previously made choice when given the chance of reevaluation. The extent to which there are interindividual differences in such processing of conflictual decisions and whether these correlate with trait anxiety remains to be established. Related to avoidance behaviors are the increased levels of intolerance to uncertainty found in anxious individuals (Beesdo‐Baum et al., 2012), which leads to negatively biased interpretations of events. Intolerance of uncertainty is linked with anger expression in individuals with generalized anxiety disorder and may explain why they may disengage from tasks or avoid decision making (Fracalanza, Koerner, Deschenes, & Dugas, 2014). It appears that intolerance to uncertainty is positively correlated with activity in frontolimbic areas, particularly in subgroups of people with social or generalized anxiety (Krain et al., 2008).

### Summary of anxiety‐related research priorities

Recent advances in anxiety neuroscience and treatment open up a number of exciting avenues for further research in decision making as discussed above: (a) How person–environment interactions influence decision‐making habits. Anxious withdrawal or irritability (Krebs et al., 2013; Mikita et al., 2015; Stoddard et al., 2014) is all potent modifiers of parental or peer responses to a child and will serve to reinforce existing decision making. The extent to which environmental modifications – as is, for example, done with standard behavioral treatment – will have an effect on the entrenched and more general decision‐making style of an anxious person has been surprisingly underexplored. (b) Attention Bias Modification Treatment (ABMT) targets a decision‐making processing in anxiety – what are its underlying neural correlates and can we use these as a guide on how to target other decision‐making processes in anxiety (such as reward processing and learning)?

## Conclusions and issues for further consideration

Children with mental health problems continually have to make decisions, yet we lack a comprehensive account of the factors and processes that may underlie decision‐making impairments in mental disorders. Important functional outcomes, including whether they can return to school, or even whether they choose to stay alive, rely on their ability to make decisions effectively. Similarly, psychological disturbances may constrain a person's capacity for decision making and impair their volition or sense of agency. While this is widely recognized in legal systems around the world, a differentiated understanding of the pathways leading to these impairments in different disorders is still lacking. In this article, we have attempted to provide an integrative, transdiagnostic neuroeconomic framework for the study of impaired decision making in psychopathology and apply it to highlight putative differences in decision making between ADHD, CD, depression, and anxiety. We argue that describing how these disorders map onto difficulties in the evaluation, execution, and/or appraisal of decisions is a key first step toward understanding why psychopathology frequently leads to negative outcomes. In this review, we have identified impairments that we predict are unique to each of the psychiatric disorders. While the focus has been on the distinct features of disorders, we acknowledge that there will also be problems cutting across current diagnostic boundaries. We have also considered the neuropsychological mechanisms that may underlie such difficulties in decision making. In general terms, there is at present only limited or indirect evidence to support these disorder‐specific hypotheses, and relevant evidence from children and adolescents is particularly scarce. Most evidence relating to decision making in childhood psychopathology comes from studies of ADHD and CD, but even here we currently lack the necessary integration across levels of analysis to establish the role of specific neurocognitive systems in driving decision‐making deficits. One consequence of this is that we have had to resort to adult studies when highlighting relevant evidence. We hope that this review will spur the field on to perform more neuroeconomically inspired studies of decision making in children with mental disorders.

Although we have addressed a wide range of issues, a number of additional issues require consideration in any discussion of decision making in child and adolescent mental disorders.

### Causality

What is the role of impaired decision making in the causal pathways from etiological factors to mental disorders? Decision making might be viewed as an expression of a disordered state – an extension of the clinical profile and a manifestation of its presentation – perhaps mediating the pathway from disorder to functional impairment and reduced quality of life. At the same time, the neuroeconomic model provides an alternative perspective on the pathophysiological pathways to disorder expression – where dysfunctional decision making associated with mental disorders appears to be a downstream effect of neural and cognitive mechanisms. For example, an overactive limbic system may bias attention toward negative stimuli, and this may in turn adversely affect the evaluation stage of decision making. Treatments such as attentional bias modification aim to reduce attentional biases toward negative stimuli and may thus improve decision making and reduce impairment. However, it is also possible that dysfunctional decision making is a causal mechanism in its own right, contributing to a vicious cycle and compounding the effects of the disorder itself. This could happen in number of ways. The decisions one makes constrain their experiences. If one chooses immediacy over delayed rewards, or safety over risk, then one reduces exposure to delay and risk and diminishes one's opportunities to learn how to manage delay and/or risk in the future. The same applies to escape from threat in anxious individuals. Decisions can also negatively impact a person's mood – that is negative predictions about the future through the process of affective forecasting can induce further hopelessness and despair in already depressed individuals. Conversely, the negative appraisal of previous decisions can impact not only on future decision making but also exacerbate negative moods and low self‐esteem. To the extent that this is true, targeting the underlying decision‐making processes may offer potential in alleviating the primary symptoms of disorders as well as associated impairment.

### Complexity, comorbidity, and heterogeneity

As mentioned in the introduction, the RDoC initiative is promoting a (reductionist) model of clinical scientific enquiry, which attempts to break mental disorders into their basic neurobiological constituent components or core impairment dimensions to provide an empirically driven transdiagnostic alternative to current clinically informed diagnostic models (Insel et al., 2010). The hope is that such an approach will progress translational science by aligning diagnostic approaches more directly to neurobiological treatment targets. In describing the complex and dynamic nature of the underlying pathophysiology of decision making in mental disorders and highlighting the ways in which basic alterations within brain systems can manifest in very different ways in different disorders, the current review highlights both of the challenges faced by researchers working within the RDoC framework. More specifically, it may only be when the dynamic interactions between brain systems or core neurocognitive processes are fully considered that the particular features of the psychopathological disorder become apparent – if this were the case, then the optimism surrounding the RDoC initiative may be misplaced. In fact, we acknowledge that the current neuroeconomic framework *underestimates* the degree of complexity of the determinants of decision making in mental disorders.

In particular, given limitations on space, two key elements contributing to such complexity have been deliberately omitted from this review. First, there is the critical issue of overlap between disorders. As a first step, we have contrasted the disorders in their archetypal and generic forms. However, we acknowledge that there is substantial overlap between the four disorders and comorbidity with other disorders (e.g. ASD) is substantial. This highlights several key questions for further consideration. For instance, if two disorders co‐occur, do their decision making attributes combine in an additive way and compound the level of impairment or does the presence of a second disorder transform the decision‐making style associated with the first? For instance, what would the combination of reckless and hesitant decision making look like in the case of the child with CD and anxiety (a far from uncommon presentation)? We have highlighted how little research directly addresses the interaction between different brain systems in decision making. However, there is even less evidence relating to comorbid presentations of disorders, although some components of our model have been investigated (i.e. executive functions in comorbid internalizing and externalizing conditions; Woltering, Lishak, Hodgson, Granic, & Zelazo, 2015). Important targets for future research are the differential characterization of introspective rumination in anxiety versus depression and how these combine in the children with both conditions. Further complexity stems from heterogeneity *within* disorders. Although earlier models have tried to map specific mental disorders onto their underlying neural substrates, it is becoming increasingly clear that psychiatric disorders are pathophysiologically heterogeneous – with different individuals with the same disorder (or at least meeting the same diagnostic criteria) showing markedly different neuropsychological profiles. This has been perhaps most fully explored in relation to ADHD (Sjowall, Roth, Lindqvist, & Thorell, 2013), where there has been a proposal for neuropsychological subtypes (Faraone et al., 2015; Nigg, Willcutt, Doyle, & Sonuga‐Barke, 2005). Heterogeneity is also considered an important issue in CD, anxiety, and depression. Accordingly, it is possible that certain subgroups within each disorder could be defined on the basis of decision‐making profiles, with some patients displaying certain impairments and others showing distinct profiles.

### Development

We have not had the space to adequately consider developmental issues. Furthermore, the limitations inherent in the present review should be acknowledged in this regard especially in relation to (a) the scarcity of child and adolescent data relating to a substantial proportion of our core hypotheses – especially for anxiety and depression; and (b) the almost complete absence of longitudinal data which would allow consideration of the developmental progression over time of decision‐making phenotypes and the underlying neurodevelopmental processes that drive changes. In thinking through developmental considerations in the future, we need to reflect on the differences between the four conditions considered here in terms of clinical and developmental profiles and timings (prodromal states, initial onset and progression, and transitions to adult life). The potential roles of neurodevelopmental immaturity and maturational delay will need to be carefully considered as contributing factors, at least in the cases of ADHD and CD (Shaw et al., 2007). Developmental models not only highlight the importance of characterizing developmental phenotypes of decision‐making across childhood and adolescence (i.e. how decision‐making profiles change with age) but also the role of the neurodevelopmental processes that shape those phenotypes. In particular, this article has concentrated on the neurobiological substrates of suboptimal decision making in mental disorders. A developmental psychopathology perspective forces us to consider the potential role of the social environment in shaping decision‐making biases or deficits.

### Social aspects of decision making

We have also not discussed other social considerations – such as the influence of social and peer processes on decision making (Smith, Chein, & Steinberg, 2014), decisions about how to treat others (e.g. whether to behave fairly or unfairly toward them), and the computations required to make sense of others' behavior (e.g. Behrens, Hunt, & Rushworth, 2009). These issues are undoubtedly important in understanding psychopathology. For example, adolescents with anxiety may be overly sensitive to social evaluation and may make suboptimal choices in an effort to conform to perceived social norms, whereas those with CD may be relatively insensitive to negative social evaluation. Initial studies suggest that adults with depression show atypical behavior in interpersonal economic exchange paradigms (Pulcu et al., 2015; Shao, Zhang, & Lee, 2015), and such tasks have shed light on abnormal social behavior in personality disorders (King‐Casas et al., 2008; Koenigs, Kruepke, & Newman, 2010). However, for reasons of space, and because there is comparatively little evidence available from studies of developmental populations, we felt that this topic should be reserved for a future review.

### Clinical implications

While it would be premature to speculate about detailed disorder‐specific clinical implications of the current review, in more general terms, a consideration of decision making raises the following clinical questions: First, would measuring decision‐making problems and related neuroeconomic parameters enhance clinicians' ability to predict a patient's overall impairment? Second, would targeting decision‐making pathology make sense in developing future treatments? As mentioned already, ABMT can be construed as such an attempt and has had variable success (Cristea, Mogoase, David, & Cuijpers, 2015). This question may be particularly relevant for treatments that are thought to affect reward processes, for example, behavioral activation in depression. Could these be usefully modified so that disturbances in decision making, rather than clinical symptoms, become the prime target? Finally, decision‐making pathology will be of particular interest to those clinicians who regularly assess their patients' capacity – a particularly underdeveloped field in child and adolescent psychiatry. Could decision‐making science provide a firmer ground for such assessments or at least be used as an additional resource?

**Key points**

- Success or failure in life is partly determined by the decisions one makes.
- Clinically, it is apparent that impaired decision‐making impacts daily functioning in young people with mental health conditions.
- To understand relationships between decision making and specific conditions, we first need to acknowledge the neuropsychological complexity of the decision‐making process – involving as it does multiple stages and neurocognitive systems.
- We propose that decision making is impaired in distinct ways in different psychopathological conditions – each reflecting a specific neurocognitive profile.
- We hypothesize that decision making is inefficient, impulsive, and inconsistent in ADHD; reckless and insensitive to negative outcomes in CD; disengaged/perseverative/pessimistic in depression; hesitant/risk‐aversive/self‐deprecating in anxiety.
- Evidence from research within a developmental psychopathology framework, across multiple levels of analysis, is required to test these hypotheses.
